# Skeletal interoception and prospective application in biomaterials for bone regeneration

**DOI:** 10.1038/s41413-024-00378-w

**Published:** 2025-01-02

**Authors:** Long Bai, Jilong Li, Guangfeng Li, Dongyang Zhou, Jiacan Su, Changsheng Liu

**Affiliations:** 1 Organoid Research Center, Institute of Translational Medicine, Shanghai University, Shanghai, China; 2 National Center for Translational Medicine (Shanghai) SHU Branch, Shanghai University, Shanghai, China; 3 Wenzhou Institute of Shanghai University, Wenzhou, Zhejiang China; 4 Department of Orthopedics, Shanghai Zhongye Hospital, Shanghai, China; 5 Department of Orthopedics, Xinhua Hospital Affiliated to Shanghai Jiao Tong University School of Medicine, Shanghai, China; 6 Key Laboratory for Ultrafine Materials of Ministry of Education, Engineering Research Center for Biomedical Materials of the Ministry of Education, East China University of Science and Technology, Shanghai, China

**Keywords:** Bone quality and biomechanics, Bone

## Abstract

Accumulating research has shed light on the significance of skeletal interoception, in maintaining physiological and metabolic homeostasis related to bone health. This review provides a comprehensive analysis of how skeletal interoception influences bone homeostasis, delving into the complex interplay between the nervous system and skeletal system. One key focus of the review is the role of various factors such as prostaglandin E2 (PGE2) in skeletal health via skeletal interoception. It explores how nerves innervating the bone tissue communicate with the central nervous system to regulate bone remodeling, a process critical for maintaining bone strength and integrity. Additionally, the review highlights the advancements in biomaterials designed to utilize skeletal interoception for enhancing bone regeneration and treatment of bone disorders. These biomaterials, tailored to interact with the body’s interoceptive pathways, are positioned at the forefront of innovative treatments for conditions like osteoporosis and fractures. They represent a convergence of bioengineering, neuroscience, and orthopedics, aiming to create more efficient and targeted therapies for bone-related disorders. In conclusion, the review underscores the importance of skeletal interoception in physiological regulation and its potential in developing more effective therapies for bone regeneration. It emphasizes the need for further research to fully understand the mechanisms of skeletal interoception and to harness its therapeutic potential fully.

## Introduction

**Fig. 1:**
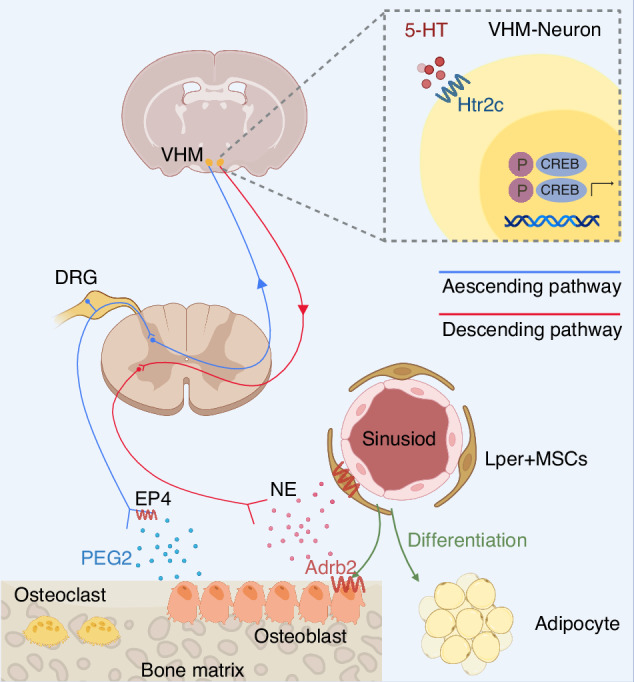
Schematic representation of the skeletal interoception

Interoception, the process through which the body perceives and responds to its internal sensory stimuli, is integral to a broad spectrum of physiological functions across multiple organ systems.^1^ For instance, in the cardiovascular system, interoception allows the body to monitor and regulate blood pressure and heart rate, ensuring hemodynamic stability.^2^ In the respiratory system, it helps regulate breathing patterns in response to changes in blood gas levels, which is vital for maintaining oxygen and carbon dioxide balance.^3^ The urinary system uses interoceptive signals to manage bladder fullness and fluid balance, directly affecting comfort and anxiety levels.^4^ Endocrine interoception monitors hormone levels, such as blood glucose, playing a key role in energy homeostasis and stress responses. Finally, skeletal interoception, although less understood, is believed to be crucial for monitoring bone health and modulating bone remodeling processes. This complex sensory system integrates both conscious and unconscious processing of these internal bodily states, making it essential not only for maintaining homeostasis but also for shaping emotional experiences and self-awareness. It plays a significant role in regulating numerous physiological responses, such as appetite, stress, and pain. In recent years, there has been a growing interest in the relationship between interoceptive mechanisms and skeletal health.^5^ Skeletal interoception specifically refers to the perception and processing of signals from the skeleton, which are essential for maintaining bone homeostasis and responding to mechanical forces.^6^ The skeletal interoceptive circuitry, encompassing sensory nerves, the central nervous system, and the sympathetic nervous system, plays a pivotal role in the precise regulation of metabolic equilibrium within the skeletal system.^7^ The discovery of skeletal interoception and its role in modulating bone remodeling has opened up a new avenue of research exploring the relationship between interoception and skeletal health.^7^ Studies have shown that bone tissue is densely and intimately innervated, suggesting that mechanical loading and other stimuli may be transmitted to the central nervous system to modulate bone remodeling.^8^ For instance, prostaglandin E2 (PGE2) secreted by osteoblasts plays a pivotal role in activating EP4 receptors located on sensory nerves, forming the ascending skeletal interoceptive pathway. Upon activation, this pathway influences the ventromedial nucleus of the hypothalamus through CREB signaling, initiated by the PGE2/EP4 interaction. This signaling cascade leads to a decrease in sympathetic nerve activity, constituting the descending interoceptive pathway. Consequently, this reduced sympathetic tone, governed by the skeletal interoceptive circuitry, prompts mesenchymal stem/stromal cells (MSCs) to differentiate into osteoblasts, thereby promoting bone formation. This intricate interplay underscores the critical role of interoceptive pathways in skeletal homeostasis and bone regeneration processes^7^ (Fig. 1). These studies have the potential to identify novel therapeutic targets for conditions such as osteoporosis, arthritis, and bone fractures, which is particularly significant as bone disorders are a significant health concern globally.

Particular, they may provide new avenues for promoting the repair of bone fractures, which currently represent a major challenge in the field of orthopedic medicine.^9^ Bone tissue engineering is an essential application of biomaterial-targeted interoceptive strategies. It involves the use of biomaterials to stimulate bone regeneration and has enormous potential for the treatment of bone defects.^10^ By utilizing biomaterials that can interact with the sensory nerves in bone tissue, it may be possible to create scaffolds that can guide bone formation and promote bone repair.^11^ Additionally, these strategies may provide new approaches for the treatment of bone infections and other chronic bone disorders. The development of biomaterial-targeted interoceptive strategies represents an exciting new direction in the field of bone research, with enormous potential for providing new therapeutic options for patients with bone disorders.^12^ However, further research is needed to determine the safety and efficacy of these new approaches, and large-scale clinical trials will be necessary to determine their clinical effectiveness.

In conclusion, this review not only elucidates the role of skeletal interoception in regulating bone homeostasis, with a particular focus on Prostaglandin E2 (PGE2) but also emphasizes the innovative design of biomaterials based on interoceptive insights. Moreover, it explores the promising frontiers of artificial intelligence and assembloids technology in the field of skeletal interoception. These cutting-edge approaches hold the potential to revolutionize our understanding and treatment of bone disorders. The future of skeletal interoception research is rich with potential, with opportunities to address key knowledge gaps and explore therapeutic applications that could lead to transformative advances in bone health. Further research is essential to translate these findings into clinically effective interventions, offering new therapeutic avenues and enhancing the efficacy of existing treatments. Large-scale clinical trials will be critical in validating these strategies, ensuring their application in improving bone health and therapy.

## Interoception

Interoception, the process of sensing the internal state of the body, is a complex and multi-faceted aspect of human physiology and psychology. It involves the interpretation and integration of signals from within the body, providing a moment-by-moment mapping of the body’s internal landscape across conscious and unconscious levels. This process is difficult to measure and manipulate experimentally in humans due to the diffuse and often unmyelinated nature of interoceptive afferents, making animal models crucial for advancing our understanding.^13^

The development of interoception as a scientific concept has been significant. Initially, it was considered to encompass senses that convey information about the body’s internal state, including tastes. However, modern definitions have expanded to include the emotional and motivational aspects of these sensations, such as the affective feelings associated with temperature regulation and the maintenance of body integrity. This broadened understanding of interoception shows that it encompasses more than visceral sensations, affecting cognitive and emotional processes, and forming the basis for subjective feelings, emotion, and self-awareness.^13^

Interoceptive signals are conveyed through a homeostatic afferent pathway, involving small-diameter primary afferents representing the physiological status of all body tissues. This system projects to autonomic and homeostatic centers in the spinal cord and brainstem, forming a direct thalamocortical representation crucial for various somatic feelings such as temperature, pain, and itch. This anatomical organization emphasizes the integral role of interoception in ongoing homeostasis, representing the physiological condition of the body itself.^14^ The lamina I spinothalamocortical pathway plays a pivotal role in interoception. For example, lamina I neurons respond selectively during and after muscle contraction, providing a substrate for the exercise pressor reflex and driving homeostatic adjustments in response to muscular work. These neurons substantiate the concept that lamina I is integral to ongoing homeostasis, relating the current physiological condition of all body tissues.^15^ This pathway’s importance is further highlighted by the fact that anterolateral cordotomy in humans, which interrupts contralateral sensations of temperature, pain, itch, sensual touch, and visceral sensations, is made at the location of ascending lamina I axons in the lateral spinothalamic tract. In primates, lamina I spinothalamic neurons project to dedicated thalamocortical relay nuclei, forming a distinct thalamocortical column orthogonal to the exteroceptive and proprioceptive representations in the ventroposterior nuclei. These findings suggest a fundamental shift in understanding pain and temperature sensations, viewing them as highly resolved sensory aspects of ongoing homeostasis.^13^

The dorsal insular cortex, activated by various interoceptive modalities, contains a primary interoceptive image of homeostatic afferents, representing several highly distinct sensations, including temperature, pain, itch, muscular and visceral sensations, and sensual touch. This region’s activation underscores the importance of the interoceptive cortex for physical well-being.^13^

Subjective awareness of feelings, a crucial aspect of interoception, is influenced by the right anterior insula and orbitofrontal cortex’s processing and re-representation. Studies using positron emission tomography and functional magnetic resonance imaging have revealed that the subjective evaluation of interoceptive stimuli depends on processing in these cortical regions. This processing supports the neurological hypothesis that the right anterior insula is integral for generating the mental image of one’s physical state, which underlies basic emotional states and is required for the motivation to make rational decisions affecting survival and quality of life. This concept aligns with the ‘somatic marker’ hypothesis of consciousness, suggesting that a refined image of the state of the body provides the basis for awareness of the physical self across time.^13^

In conclusion, interoception plays a critical role in a wide range of human functions, from gathering and removing resources for energetic needs to shaping our sense of self and emotional experiences. It involves complex neural pathways and regions, particularly the lamina I spinothalamocortical pathway and the dorsal posterior insula, underscoring its fundamental importance in the physiological and psychological aspects of human life.

## Skeletal interoception

The skeletal system, being one of the largest organs in the body, plays a fundamental role not only in supporting terrestrial movement but also in regulating whole-body metabolism. It is extensively innervated by sensory nerves that facilitate the transmission of mechanical and biochemical signals to the CNS, a process known as skeletal interoception. This neural circuitry allows the brain to perceive mechanical loading and gravitational forces, which in turn modulates bone remodeling and homeostasis. The interaction between sensory innervation and the skeletal system is vital for maintaining bone health, especially in response to external stimuli like mechanical stress.^16^ This dynamic regulation is closely linked to both the structural role of the skeleton and its endocrine functions, influencing calcium, phosphate storage, and overall metabolic balance.

From the perspective of brain-to-bone communication, neurological factors such as chronic stress, depression, and neurodegenerative diseases significantly affect bone health.^17^ These conditions increase pro-inflammatory cytokines and disrupt hormonal balances, leading to elevated bone resorption and higher fracture risks. Conditions like Alzheimer’s disease, Parkinson’s disease, and traumatic brain injuries are associated with reduced bone mineral density and impaired fracture healing, showing that the brain’s influence on bone health is profound and multifaceted. The CNS plays a critical role in regulating bone metabolism through neuroendocrine signals and sensory feedback, further highlighting the importance of the brain-bone axis in overall skeletal health.^18^

Conversely, the feedback loop between bone and the brain is equally significant.^17^ Bone diseases and injuries, such as osteoporosis, fractures, or complex regional pain syndrome, can affect the CNS through altered sensory signaling. These conditions disrupt neural homeostasis, leading to abnormal neuroplasticity, chronic pain, and metabolic imbalances that impact cognitive function and emotional states. The complex interactions between bone and brain are essential not only for physical health but also for maintaining neurological and systemic metabolic functions. Understanding these reciprocal interactions through skeletal interoception paves the way for novel therapeutic approaches in treating bone disorders and neurodegenerative diseases, providing a comprehensive framework for addressing both bone and brain health simultaneously.

## Factors regulated skeletal interoception

The factors involved in skeletal interoception encompass a range of bioactive substances that are integral in the communication between the nervous system and the skeletal system. These factors include neurotransmitters and neuropeptides, axon guidance factors, and neurotrophins, each playing unique roles in this complex interplay (Table 1).

**Table 1:** Factors involved in skeletal interoception

Bioactive factors		Roles of peripheral nervous system	Roles of skeleton
Neurotransmitters and Neuropeptides	NE	Synthesis and secretion NE: secreted by sympathetic adrenergic nerves^76^	Regulation (1) enzymes MAOa and MAOp: expressed in osteoblast precursor cells and in fully differentiated osteoblasts^77,78^ (2) NE transporter: expressed in differentiated osteoblasts^77^
	ACh	Synthesis and secretion ACh: secreted by cholinergic nerves^79^	Regulation (1) VAChT, the choline transporter, and CarAT: expressed in osteoblasts^80,81^ (2) AChE and BChE: expressed in osteoblasts^80,81^
	CGRP	Synthesis and secretion CGRP: secreted by sensory nerves^82^	Regulation CGRP: expressed in human osteosarcoma cells and primary osteoblasts^83^
	VIP	Synthesis and secretion VIP: secreted by cholinergic nerves^84,85^	Regulation VIP: expressed in isolated pure populations of osteoclasts^86^
	NPY	Synthesis and secretion NPY: secreted by sympathetic adrenergic nerves^87^	Regulation NPY: produced by osteocytes and osteoblasts^88^
	SP	Synthesis and secretion SP: secreted by sensory nerves^89,90^	Regulation SP and the receptor NK-1: expressed in osteoblasts and osteocytes especially under mechanical stimulation during exercise^91^
Axon guidance factors	Sema3A	Synthesis and secretion Sema3A: secreted by a specific subset of nerves including sensory nerves^92^	Synthesis and secretion Sema3A: secreted by osteoblast lineage cells and expressed in bone cell lineages including chondrocytes, osteoblasts, and osteoclasts^93,94^
	Sema4D	Synthesis and secretion Sema4D and its receptors plexin-B1, plexin-B2: expressed in embryonic dorsal root ganglion^95^	Synthesis and secretion Sema4D: strongly expressed in osteoclasts, with no evidence of its expression in osteoblasts^96–99^
	Netrin-1	Synthesis and secretion Netrin-1: continuously expressed in the nervous system. Expression in SCs upregulated during nerve repair^100^	Synthesis and secretion Netrin-1: produced by osteoblasts and osteoclast. Expression of Netrin-1 in osteoblasts was found to be 200-fold higher than that in osteoclasts^101–103^
	Slit-3	Synthesis and secretion Slit-3: expressed in the cell bodies and axons of both motor and sensory neurons, satellite cells of the dorsal root ganglion, SCs and fibroblasts of peripheral nerves^104^	Synthesis and secretion Slit-3: secreted by osteoclasts and osteoblasts. Production increasing during osteoclast differentiation^105,106^
Neurotrophins	NGF	Synthesis and secretion NGF: highly concentrated in the nervous system during nerve development or regeneration. Secreted by SCs of peripheral nerves^107^	Synthesis and secretion NGF: expressed in bone marrow stromal cells, osteoblasts as well as osteoblastic cell lines. Expression of NGF is upregulated during proliferation or upon loading^108–110^
	BDNF	Synthesis and secretion BDNF: highly concentrated in the nervous system during nerve development or regeneration. Secreted by SCs of peripheral nerves^107^	Synthesis and secretion BDNF and its receptor TrkB: expressed in fracture bone tissues during early bone formation. Concentrated in endothelial and osteoblastic cells^108,111,112^

### Neurotransmitters and neuropeptides

These include norepinephrine (NE), acetylcholine (ACh), calcitonin gene-related peptide (CGRP), vasoactive intestinal peptide (VIP), neuropeptide Y (NPY), and substance P (SP). NE, secreted by sympathetic adrenergic nerves, is regulated by enzymes and transporters expressed in osteoblasts. ACh, produced by cholinergic nerves, influences osteoblasts through various transporters and enzymes. CGRP and VIP, secreted by sensory and cholinergic nerves respectively, have regulatory roles in osteosarcoma cells, osteoblasts, and osteoclasts. NPY and SP, both secreted by sensory and sympathetic nerves, are involved in the regulation of osteocytes and osteoblasts, particularly under mechanical stimulation.

### Axon guidance factors

These include Semaphorin 3A (Sema3A), Semaphorin 4D (Sema4D), Netrin-1, and Slit-3. Sema3A, secreted by specific subsets of nerves, is also produced by various bone cell lineages. Sema4D is expressed in osteoclasts but not in osteoblasts. Netrin-1, continually expressed in the nervous system, is predominantly produced by osteoblasts. Slit-3, found in both motor and sensory neurons, is secreted by osteoclasts and osteoblasts, with increased production during osteoclast differentiation.

### Neurotrophins

These include nerve growth factor (NGF) and brain-derived neurotrophic factor (BDNF). NGF, concentrated in the nervous system during development and regeneration, is also expressed in bone marrow stromal cells and osteoblasts, with upregulated expression during proliferation or loading. BDNF, along with its receptor TrkB, is expressed in fracture bone tissues during early bone formation, particularly in endothelial and osteoblastic cells.

In summary, these factors collectively facilitate skeletal interoception by bridging the nervous and skeletal systems. They regulate various aspects of bone metabolism, including osteoblast differentiation, bone mass accrual, and response to mechanical stress, underscoring their significance in maintaining skeletal health and integrity.

## PGE2 mediated skeletal interoception

PGE2 is a bioactive lipid with a broad spectrum of physiological roles facilitated by its interaction with the E-type prostanoid (EP) receptor family. Its biological activities are crucial for the regeneration of various organ systems, particularly post-injury, leveraging processes like the activation of endogenous stem cells, immune system modulation, and the promotion of angiogenesis.^19^ PGE2 has also been implicated in the pathophysiology of cancer, specifically in the progression of hepatocellular carcinoma. It can promote the proliferation and migration of liver cancer cells, affecting hepatocytes and the tumor microenvironment through various signaling pathways, including ERK/COX-2/PGE2 in hepatic stellate cells.^20^

Moreover, PGE2 plays a modulatory role in several physiological responses such as inflammation, fever, and muscle regeneration, and has been linked to skeletal muscle function through patents related to its application in this area.^21^ In the cardiovascular system, PGE2 is among the most abundant bio-active lipids, where it exerts significant effects on vascular tone, cardiac remodeling, and inflammatory processes. Its influence on these biological functions highlights the extensive impact of PGE2 on systemic health and disease.^22^ These diverse roles underscore PGE2’s importance in bodily functions and its potential as a therapeutic target. In recent years, PGE2 is not only implicated in pain perception and inflammation associated with musculoskeletal conditions but also in the complex regulation of bone metabolism. This regulatory capacity of PGE2 is especially apparent in its interplay with sensory nerves, which forms a cornerstone for skeletal interoception, subtly influencing bone remodeling and integrity.

### Skeleton interoception maintains bone homeostasis

**Fig. 2:**
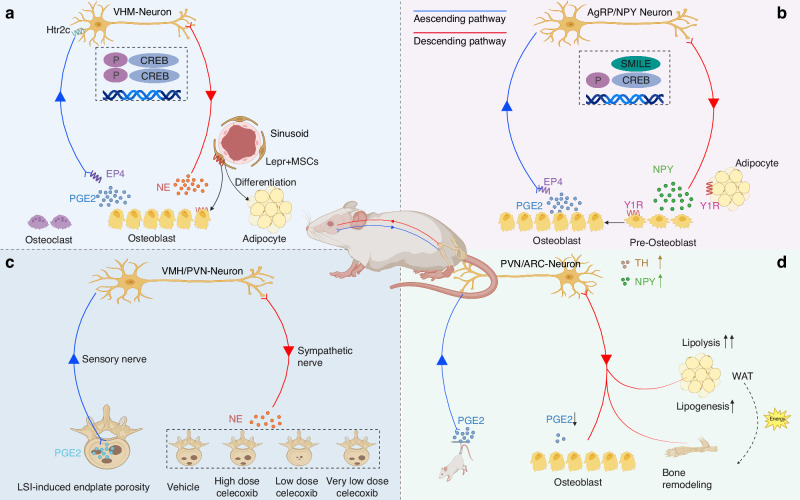
The role of PGE2 in skeletal interoception. **a** Skeleton interoception maintains bone homeostasis via PGE2. Reprinted with permission.^23^ **b** Skeleton interoception balance metabolism between bone and fat via PGE2. Reprinted with permission.^7^ **c** Skeleton interoception-induced modulation of endplate porosity via PGE2. Reprinted with permission.^16^ **d** Skeleton interoception modulation under microgravity conditions via PGE2. Reprinted with permission^13^

The role of PGE2 in skeletal interoception is multifaceted, influencing both the sensory nervous system and MSC differentiation. PGE2, secreted by osteoblasts, activates the sensory nerve EP4 receptor, which in turn modulates bone formation by inhibiting sympathetic nerve activity. This regulatory axis is crucial for maintaining bone homeostasis, as evidenced by studies demonstrating that disruptions in this pathway lead to significant shifts in MSC differentiation towards adipogenesis at the expense of osteogenesis. Furthermore, interventions that manipulate PGE2 levels or sympathetic tone can significantly influence MSC fate decisions, emphasizing the PGE2/EP4 sensory nerve axis as a potential therapeutic target for bone-related conditions^23^ (Fig. 2a).

Bone homeostasis is maintained through a delicate balance of osteoblast, adipocyte, and chondrocyte differentiation from MSCs. Sensory nerves play a pivotal role in this process by sensing the “bone-forming signal” PGE2 and modulating the activity of sympathetic nerves. The discovery of this interaction between PGE2 and sensory nerves provides novel insights into the skeletal system’s complexity and underscores the potential of targeting the PGE2/EP4 axis in developing treatments for bone disorders. Additionally, the deletion of the EP4 receptor in sensory nerves mirrors the effects observed in sensory denervation models, further highlighting the essential role of this receptor in bone formation and MSC differentiation. The EP4 receptor is part of the PGE2 receptor family, which includes four receptors (EP1-EP4), each playing a part in PGE2’s broad biological functions. The research on EP4’s role in skeletal interoception thus opens up new avenues for understanding and potentially intervening in bone metabolism and related diseases.

### Skeleton interoception balance metabolism between bone and fat

PGE2 activated by osteoblasts, interact with the EP4 receptor on sensory nerves, precipitating a cascade of effects that culminate in the modulation of bone remodeling and fat metabolism.^24^ One of the pivotal findings is the role of PGE2 in downregulating hypothalamic neuropeptide Y (NPY), which induces lipolysis in adipose tissue that is essential for osteoblastic bone formation. This process is mediated through the induction of SMILE expression in the hypothalamus, which then represses NPY expression by binding to its promoters. Disruption of this pathway, such as through the knockout of EP4 in sensory nerves, results in an increase in NPY, leading to bone catabolism and enhanced fat accumulation (Fig. 2b). Furthermore, the inhibition of the NPY Y1 receptor has been shown to promote the oxidation of free fatty acids in osteoblasts, aiding in the reversal of bone loss conditions in experimental models. The study underscores the complex interplay between neuroendocrine signals and cellular metabolism that orchestrates the balance between bone formation and adiposity, highlighting the translational potential of targeting skeletal interoceptive pathways in treating metabolic bone diseases.

### Skeleton interoception-induced modulation of endplate porosity

It has been identified that PGE2, secreted by osteoblasts, activates the EP4 receptor on sensory nerves, leading to the phosphorylation of cAMP response element-binding protein (CREB) in the hypothalamus, thus influencing sympathetic activity and promoting osteoblastic bone formation. The findings suggest that maintaining physiological levels of PGE2 is crucial for skeletal interoception, which regulates bone density, mechanical stress response, and metabolic activity in bone tissue.

A recent study delves into the impact of PGE2 concentration on vertebral endplate porosity and spinal pain, revealing that high PGE2 levels are associated with increased pain, particularly in conditions like osteoarthritis and spinal degeneration.^16^ Notably, the application of low-dose celecoxib successfully maintained PGE2 at physiological levels, leading to significant reductions in endplate porosity and spinal pain, an effect that remained even after the cessation of treatment (Fig. 2c). This effect of low-dose celecoxib was mediated through the modulation of skeletal interoception, as knockout of the EP4 receptor in sensory nerves negated the drug’s beneficial effects. Overall, the study underscores the potential of utilizing low-dose celecoxib to manage PGE2/EP4-mediated skeletal interoception, offering a novel approach to reducing vertebral endplate porosity and managing chronic spinal pain without impairing bone formation, providing a promising therapeutic avenue for skeletal diseases.

### Skeleton interoception modulation under microgravity conditions

A most recent study found that decreased PGE2/EP4-mediated signaling due to unloading induces TH and NPY expression in the hypothalamus.^25^ These changes increase sympathetic tone, which then stimulates osteocyte-mediated bone resorption and white adipose tissue lipolysis. Conversely, inhibiting sympathetic output, or the action of hypothalamic NPY, mitigates bone loss and modifies fat metabolism in the model of unloaded mice, showcasing the significant role of PGE2/EP4-driven skeletal interoception in these processes (Fig. 2d). Interventions that block the sympathetic activity, such as the use of the antagonist propranolol, and osteocyte-specific deletion of Adrb2, attenuated bone loss, indicating the involvement of sympathetic signaling in bone remodeling under unloading conditions. The study also demonstrates that depleting sympathetic nerves or blocking norepinephrine (NE) release can prevent bone loss, highlighting the regulatory role of sympathetic function in skeletal interoception and bone resorption during unloading.

Additionally, the elevation of hypothalamic NPY was found to regulate fat metabolism during unloading, indicating that NPY is a significant neuroendocrine factor controlling lipogenesis and the balance between bone and fat metabolism. This regulation of fat and bone metabolism facilitates interoceptive TH-regulated bone remodeling under simulated microgravity conditions. Accordingly, the study delineates the intricate PGE2/EP4-mediated pathways that govern skeletal interoception and underscores how alterations in these pathways under conditions of microgravity can lead to changes in bone density and fat metabolism. The data presented emphasize the importance of PGE2 as a key factor in the skeletal interoceptive signaling network, particularly in the context of spaceflight and other unloading conditions.

In summary, PGE2 stands out as a potent regulator within the skeletal interoception network, with its effects extending from cellular to systemic levels. Its modulation offers promising therapeutic strategies for enhancing bone regeneration and treating bone-related disorders, highlighting the importance of further research to harness its full therapeutic potential.

## Biomaterials strategy based on skeletal interoception for bone regeneration

The intricate role of the nervous system in orchestrating bone regeneration is nothing short of remarkable.^26^ Not only does it provide essential sensory and motor functions for our physical well-being, but it also acts as a dynamic regulator in the bone remodeling process. Recognizing the pivotal role of the nervous system in bone repair, researchers have turned to innovative biomaterial strategies.^27^ By developing bioengineered materials that interact with and guide neural components, they aim to further enhance bone repair processes.^28^ To achieve this, the development of these biomaterials relies on the integration of specific modules, each playing a pivotal role in guiding neural growth and influencing bone regeneration processes.

### Biomaterial matrix

When constructing biomaterials for guiding neuro-regulation in bone repair, the material’s matrix should exhibit several essential characteristics. First and foremost, it must be biocompatible, ensuring compatibility with the host organism to minimize inflammation and immune responses while facilitating cell adhesion and tissue integration.^29^ Biodegradability is equally important, allowing the material to be gradually replaced by newly formed tissue as the healing process unfolds.^30^ To provide structural support during regeneration, the material should also maintain stability under mechanical stress.^31^ Hydroxyapatite (HA), for instance, stands out for its biocompatibility, closely mimicking the mineral phase of natural bone and providing a solid scaffold for bone regeneration.^32^ Polycaprolactone (PCL), a biodegradable polymer, is renowned for its mechanical strength and stability, making it an ideal framework for bone repair while also offering the flexibility to release neuroregulatory cues gradually.^33^ Poly (lactic-co-glycolic acid) (PLGA) is another versatile biodegradable polymer that can be engineered to degrade at controlled rates and can have its surfaces modified to support neural cell growth.^34^ Some biomaterials inherently possess neuroregulatory and osteogenic properties, stimulating neural and bone tissue growth, and these properties can be harnessed to facilitate interactions between neural cells and the material. For example, natural polymers like chitosan, derived from chitin, exhibit unique properties, including biocompatibility and the ability to stimulate neurogenesis.^35,36^ These can be tailored to incorporate growth factors and provide a suitable substrate for neural tissue. Collagen, a major component of the extracellular matrix, offers excellent biocompatibility and the capacity to be enriched with bioactive molecules, rendering it an ideal candidate for supporting the growth of both bone and neural tissue.^37,38^ These biomaterials collectively embody the characteristics necessary for neuro-regulation in bone repair, making them well-suited for facilitating effective bone tissue regeneration within the domain of regenerative medicine.

### Neuroregulatory factors

As mentioned above, the close interplay between the nervous system and bone homeostasis can be significantly leveraged in bone tissue regeneration by supplementing with exogenous neuropeptides,^39^ brain signaling proteins,^40^ and hormones,^41^ which all hold the potential to markedly enhance the repair and regeneration of bone tissues. Therefore, the incorporation of these bioactive factors is a crucial aspect in material construction for guiding neuro-regulation in bone repair. This strategy is substantiated by the intricate connections between neural regulation and bone repair.^42^ For instance, neuropeptides like substance P have been found to play a vital role in modulating bone metabolism by regulating osteoblast and osteoclast activities.^43^ Similarly, BDNF, a prominent brain signaling protein, has been shown to exert a positive influence on bone density.^44^ Furthermore, hormones such as estrogen are well-documented for their effects on bone health, and their supplementation in bioactive materials can provide a conducive environment for enhanced bone regeneration.^45^ By integrating these bioactive factors into material design, we can harness the power of neuro-regulation to optimize the repair and regeneration of bone tissues.

### Cell implantation

Cell implantation is a prominent avenue for the neural regulation of bone tissue repair. Several cell types can effectively modulate bone tissue repair through their interaction with neural tissue. For instance, SCs,^46^ mesenchymal stem cells,^47^ osteoblasts,^48^ and sensory neurons^49^ play crucial roles. The choice of these cells is often based on their specific characteristics and functions within the neural and bone microenvironment. For example, Schwann cells are known for their ability to produce neurotrophic factors and support neural regeneration.^11^ Mesenchymal stem cells offer a versatile source of regenerative potential, capable of differentiating into various cell types required for tissue repair.^50^ Osteoblasts contribute directly to bone formation, while sensory neurons play a role in relaying important sensory information and promoting tissue regeneration. The method through which these cells exert their influence involves their interaction with the neural environment, releasing neurotrophic factors and other signaling molecules. These signaling molecules facilitate crosstalk between neural and bone tissues, ultimately promoting bone repair.

### 3D structural design

The construction of the biomaterial’s three-dimensional architecture is a meticulous process aimed at providing an optimal environment for neural guidance and bone repair. This involves the incorporation of micro/nano-topographical features, porous structures, and precisely designed channels intended to facilitate the extension of neurites, the projection of neurons.^51^ These structural elements serve the purpose of orchestrating and directing the growth of neurons in a controlled manner, ensuring that they follow specific paths and become aligned with precision. The micro/nano-topographical features offer a structured landscape where neurons can navigate effectively. These features provide physical cues to guide neurites and promote their growth along desired routes. Porous structures within the biomaterial not only enhance the integration of cells but also create passageways for neurons to traverse.^52^ These pathways ensure that the neural network establishes itself within the material. Moreover, the designed channels within the biomaterial serve as dedicated conduits for neurites to follow. These pathways are strategically placed to encourage neural extensions in specific directions, aligning with the overall objective of neural guidance. As a result, the three-dimensional architecture plays a pivotal role in fostering the interaction between neural and bone tissues, ultimately promoting effective neural regulation of bone repair. This precise engineering of the biomaterial’s structure is paramount in advancing the field of regenerative medicine and enhancing the success of neural-guided bone repair.

### Controlled releasing system

In the pursuit of directing neural growth for the enhancement of bone repair, controlled release systems emerge as indispensable tools. These systems are designed to encapsulate or administer a repertoire of neuroregulatory factors and growth factors in a meticulously controlled and sustained fashion.^10^ This strategic approach guarantees that vital signaling molecules are dispensed with precision, ensuring they are available at the precise moment and location necessary for steering neural development and facilitating bone healing. The controlled release systems act as sophisticated delivery vehicles, intricately designed to regulate the release kinetics of these bioactive factors. By encapsulating these neuroregulatory and growth factors within the matrix of the biomaterial, the controlled release system enables them to be discharged at an optimal rate.^53^ This controlled and sustained release ensures that the signaling molecules are available in an orchestrated manner, aligning with the dynamic needs of neural guidance and bone repair. This approach is instrumental in achieving the desired outcome: to create a microenvironment within the biomaterial that promotes both neural and bone tissue interactions. It orchestrates the release of signaling molecules precisely where they are needed, stimulating neural growth along predetermined pathways and contributing to effective bone healing.^54^ The marriage of controlled release systems with biomaterials is a critical strategy, advancing the field of regenerative medicine and underscoring its potential to revolutionize neural-guided bone repair.^55^

## Application of skeletal interoception in biomaterials for bone regeneration

The human skeletal system is innervated by neural fibers that connect the dorsal root ganglia with the central nervous system. Neural fibers are distributed throughout the entire skeletal structure, including the periosteum, bone marrow, and the mineralized portions of bones. Therefore, the normal functioning of the nervous system is crucial for maintaining bone homeostasis and the healing of fractures. The following section will focus on the specific applications of innervated biomaterials in bone.

**Fig. 3:**
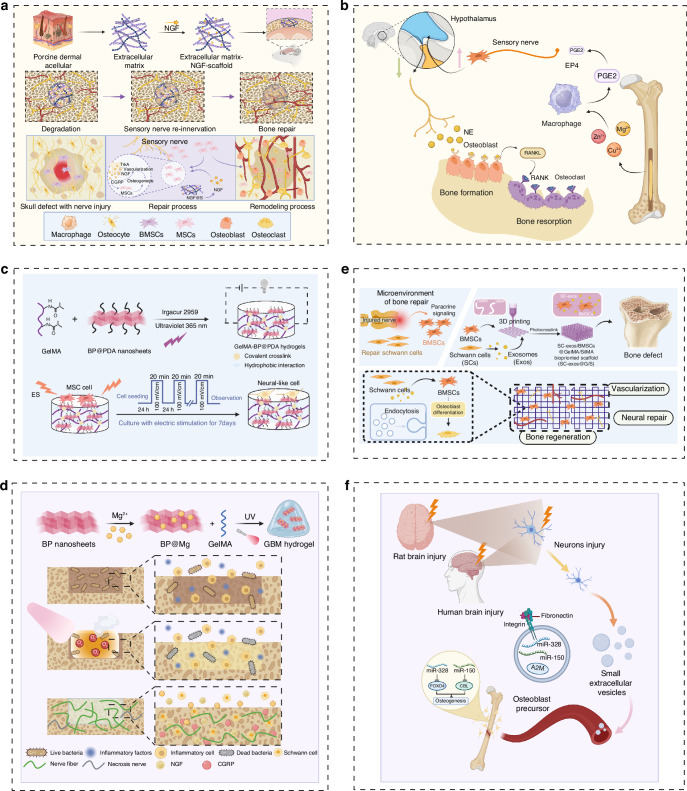
**a** Engineered sensory nerve guides self-adaptive bone healing via NGF-TrkA signaling pathway. Reprinted with permission.^59^ **b** Schematic diagram showing the process of divalent metal cations participate in the regulation of bone formation through interoceptive signaling. Reprinted with permission.^1^ **c** Incorporation of the polydopamine-modified black phosphorus nanosheets (BP@PDA) into GelMA network (up). Schematic diagram depicting the MSCs growth and differentiation on conductive GelMA-BP@PDA hydrogel with the assistance of external electrical stimulation(down). Reprinted with permission.^64^ **d** Schematic diagram of the GelMA-BP@Mg (GBM) hydrogel for antibacterial and innerved bone regeneration of infected bone defects. Under NIR irradiation, the GBM hydrogel has high antibacterial efficiency and reduces the damage of bacteria to bone tissue. Magnesium-modified black phosphorus (BP@Mg) nanosheets promoted skeletal-associated nerve fiber repair and accelerated bone regeneration. Reprinted with permission.^65^ **e** Schematic illustrating the procedures used to cross-link bioprinted constructions, which can improve the bone regeneration microenvironment to promote neurovascularized bone repair. Reprinted with permission.^11^ **f** Scheme of the mode of damaged brain accelerates bone healing by releasing sEVs that target osteoprogenitors. Reprinted with permission^12^

Innervation serves as the initiating factor for bone regeneration and plays a crucial regulatory role in subsequent processes including vascularization, ossification, and mineralization. The most common approach is to load NGF, neurotrophic peptides, and other nerve growth-promoting substances into carriers such as hydrogels, thereby promoting the bone repair process. For example, NGF-carrying collagen/nano-hydroxyapatite/alginate hydrogel has been proved to have good osteogenic effect in mandibular traction.^56^ The research suggested that a decrease in the count of sensory nerve fibers in individuals with osteoporosis, coupled with an increase in sympathetic nervous activity, underscoring the vital contribution of neural innervation to bone health. In the fields of embryology and traumatology, sensory nerve fibers are primarily located in metabolically active regions and participate directly in osteogenesis through the secretion of neuropeptides. CGRP and SP are the most representative neuropeptides involved in osteogenesis. Histological analysis demonstrates that gelatin microspheres containing CGRP or SP effectively promote bone formation in osteoporotic rabbits when loaded into the microspheres for sustained release.^57^Titanium implants coated with graphene oxide (GO) serve as carriers for delivering SP and Bone Morphogenetic Protein 2, demonstrating a similar ability to enhance bone formation.^58^ Finally, Yu et al. utilized porcine decellularized dermal matrix to prepare a decellularized extracellular matrix (dECM). They created an NGF-releasing system by non-covalently binding NGF to the nanofiber scaffold of dECM. This system took advantage of dECM’s natural porous structure and binding sites for various growth factors. In vitro experiments demonstrated enhanced differentiation and neurite outgrowth of SCs and rat dorsal root ganglions (DRGs), along with increased expression of neural-bone crosstalk and CGRP. The BMSCs/DRGs/scaffold co-culture model showed more significant promotion of BMSC osteogenic differentiation than the control group, mediated through the CGRP-cAMP-CREB signaling pathway. In vivo experiments revealed sustained release of NGF@S induced engineered sensory nerve innervation, effectively promoting new bone formation and vascularization (Fig. 3a).^59^

Magnesium is an essential and vital element for maintaining optimal bone health. Its significance in promoting strong and healthy bones cannot be overstated. In the field of orthopedics, innovative biodegradable implants made from magnesium have emerged as a transformative solution for bone fracture repair. These implants, crafted from biodegradable magnesium materials, represent a pioneering approach to not only mending fractures but also facilitating the regeneration of robust and resilient bone tissue. However, the potential mechanisms behind the improved bone healing facilitated by these biodegradable magnesium implants remain largely uncharted. Qin et al. conducted an experiment where they implanted intramedullary rods containing ultra-pure magnesium into the intact distal femurs of rats. This procedure resulted in the formation of new bone tissue around the cortical bone of the femur. Upon investigating the mechanisms at play, it was discovered that the increased formation of new bone was accompanied by a significant upregulation of Calcitonin Gene-Related Peptide-alpha (CGRP-α) in the periosteum of the femur and the DRG on the same side. In vivo, experiments involving the downregulation of CGRP receptor-encoding genes showed that magnesium-induced osteogenesis was inhibited. Conversely, overexpression of CGRP receptor genes enhanced the osteogenic effect induced by magnesium. This research was further validated using a rat femoral fracture model induced by ovariectomy-induced osteoporosis. Implants containing magnesium were inserted into the medullary cavity of the femur, providing additional evidence of magnesium’s role in promoting neural-mediated osteogenic differentiation.^60^ A subsequent experiment further underscored the clinical potential of magnesium implants in orthopedics. Implanting magnesium nails into the bone marrow cavity not only promoted osteogenesis but also facilitated angiogenesis. Blocking sensory nerves with capsaicin slowed down the bone defect repair process. Furthermore, the insertion of magnesium nails upregulated the expression of CGRP in a concentration-dependent manner. This upregulation of CGRP promoted endothelial cell migration and tube formation. CGRP also stimulated the phosphorylation of the adhesion plaque kinase at the Y397 site (FAK) and the expression of vascular endothelial growth factor (VEGF). This suggests that the CGRP-FAK-VEGF signaling axis linking sensory nerves to endothelial cells is likely a key mechanism by which magnesium participates in bone defect repair.^61^ Apart from magnesium, there is scientific evidence suggesting that silicon also plays a role in promoting bone regeneration and repair by triggering sensory nerves to produce neuropeptides. In a study conducted by Niu et al., a silicified collagen scaffold was implanted into a rat model with femoral defects, resulting in an accelerated repair of the bone defect. In vitro experiments demonstrated that culturing mesenchymal stem cells and endothelial progenitor cells in the conditioned medium of DRG cells, stimulated by silicon, promoted their differentiation. This promoting effect was found to be inhibited by neutralizing antibodies against Sema3A. Additionally, in a rat model of femoral defect, knocking down Sema3A in DRGs almost completely impeded silicon-induced bone and vascular formation, while overexpression of Sema3A facilitated silicon-induced phenomena. These findings affirm the involvement of silicon in inducing the production of Sema3A in sensory nerves, thereby stimulating osteogenesis and angiogenesis.^62^ The outlined information suggests that metal ions play an inductive role in bone formation. In a conducted study by Cao et al., divalent metal cations, such as Mg^2+^, Zn^2+^ and Cu^2+^, were found to activate the immune-neural axis. This activation induced responses within the skeletal system and initiated the central nervous system’s regulatory mechanisms for bone formation. Notably, the emergence and branching of sensory nerves, stimulated by macrophage-derived prostaglandin E2, convey interoceptive signals to the central nervous system, modulating sympathetic activity through hypothalamic CREB signaling. This, in turn, leads to enhanced osteogenesis and reduced osteoclastogenesis in the injured bone (Fig. 3b).^1^ This explains the intrinsic mechanisms through which divalent metal ions promote bone formation.

Conductive hydrogel scaffolds have vital roles in mending electroactive tissues. Electric signals produced by endogenous electric fields play a vital role in the process of tissue repair during both skeletal and neural regeneration. The transmission of these signals can be enhanced using conductive materials. Black phosphorus (BP) exhibits significant promise in the treatment of infectious bone defects owing to its photothermal and photodynamic characteristics. Experiments have provided evidence that black phosphorus nano scaffolds can induce angiogenesis and neurogenesis under conditions of mild oxidative stress.^63^ With its remarkable electrical conductivity, BP exerts a beneficial influence on the repair of neural tissues and the regeneration of bone. Luo et al. have introduced a biodegradable, conductive hybrid hydrogel that integrates BP nanosheets into the hydrogel matrix. After modification with dopamine, this hydrogel notably enhances the differentiation of MSCs into neuron-like cells (Fig. 3c).^64^ In addition, their integration of BP hydrogels with photothermal therapy and magnesium led to the development of a photosensitive conductive hydrogel known as GelMA-BP@Mg. The released conductive nanosheets from BP@Mg, working in synergy with biologically active ions, significantly improved Schwann cell migration and secretion. This promoted neurite growth and advanced the regeneration of both nerve and bone tissues. Notably, in a skull defect model with infection, the GelMA-BP@Mg hydrogel demonstrated effective antibacterial activity while facilitating the regeneration of bone and CGRP nerve fibers (Fig. 3d).^65^

Extracellular vesicles (EVs) released by nerve cells have intriguingly demonstrated the potential to stimulate osteogenesis. Comprising a phospholipid bilayer, proteins, and nucleic acids, EVs play a pivotal role in intercellular communication. Wang et al. initially established that SCs, a principal cellular component of the peripheral nervous system, modulate the microenvironment to facilitate the growth and osteogenic differentiation of BMSCs through exosomes. Subsequent work utilized bioprinting to incorporate BMSCs and Schwann cell-derived exosomes into GelMA and SilMA hybrid hydrogels (SC-exos@G/S), printed into specific structures. Implantation of this engineered construct beneath rat skin and within a rat skull defect model substantiated the potency of SC-exos@G/S in enhancing vascularization and bone regeneration (Fig. 3e).^11^ Furthermore, small extracellular vesicles (sEVs) originating from damaged neurons have shown the capacity to stimulate bone formation. Bai et al. made an intriguing discovery, finding that plasma-derived sEVs from traumatic brain injury patients and rats possess the potential to enhance osteogenesis, even surpassing the effects of soluble factors. Investigating the origin, these osteogenic sEVs, originating from injured neurons during traumatic brain injury, target the skeletal system and osteoprogenitor cells, promoting osteogenesis, which is attributed to the microRNAs carried by the sEVs. In vivo experiments demonstrate that hydrogels loaded with these sEVs effectively promote bone formation and repair bone defects (Fig. 3f).^12^

Finally, to develop an implant material that can promote the formation of both neural and bone tissue, autologous nerve grafts are typically integrated into biomaterials. For example, Pei et al. conducted research demonstrating the effectiveness of implanting sensory nerve bundles to repair substantial bone defects. In their subsequent studies, they introduced BMSCs into β-tricalcium phosphate scaffolds. They then performed a distal transection of the sciatic nerve, removed the distal part of the nerve sheath, separated the nerve bundles, and inserted the isolated sciatic nerve into the lateral grooves of the β-tricalcium phosphate scaffold. This allowed the nerve bundles to grow through the scaffold’s porous structure. Neuro-tracing techniques confirmed that the implanted sensory nerve bundles reached the scaffold pores ahead of blood vessels. The experimental results revealed a notable increase in the expression of CGRP during the bone defect healing process when compared to the control group.^66,67^

In conclusion, the utilization of innervated bone biomaterials represents a groundbreaking approach in the realm of skeletal tissue regeneration (Table 2). By intricately integrating the principles of neural guidance with cutting-edge biomaterial technologies, researchers aim to revolutionize the landscape of bone repair and regeneration. The symbiotic relationship between the nervous system and bone health is accentuated through the development and application of these innovative materials. From conductive hydrogel scaffolds to the incorporation of black phosphorus nanoscaffolds, the repertoire of materials designed for neuro-regulation in bone repair is expanding. Moreover, the role of essential elements like magnesium and silicon, alongside the potential of extracellular vesicles derived from damaged neurons, adds layers of complexity to the strategies employed. As we delve into the intricacies of innervated bone biomaterials, we uncover promising avenues for enhancing not only the structural integrity of bone but also the dynamic interplay between neural networks and regenerative processes. This marks a pivotal chapter in the ongoing quest for advanced therapeutic interventions that hold the promise of revolutionizing skeletal tissue regeneration.

**Table 2:** Application of skeletal interoception in biomaterials for bone regeneration

Materials	Bioactive factors	Characteristics	Functions	Ref.
Collagen/nano-hydroxyapatite/alginate hydrogel	NGF	Injectability and sustained release ability	Improve bone formation	^56^
Gelatin microsphere	CGRP or SP	Promote osteogenesis	Repair bone defect	^57^
Graphene oxide-coated titanium	BMP-2 and SP	Promote MSC recruitment and osteogenesis	Improve bone formation	^58^
dECM	NGF and BMP‐2	Via NGF‐TrkA signaling pathway and CGRP‐dependent mechanism	Optimize bone homeostasis	^59^
Magnesium-containing intramedullary nail	Ultrapure magnesium	Increase the expression of CGRP and ATP；	Improve the osteogenic differentiation and bone-fracture healing;	^58,60^
Magnesium-containing intramedullary nail	Ultrapure magnesium	Via CGRP-FAK-VEGF signaling axis	Stimulate bone tissue regeneration	^59,61^
Silicified collagen scaffold	Silicon	Induces semaphorin 3A secretion by sensory nerves	Improve in-situ bone regeneration	^62^
Alginate-based hydrogel	Mg^2+^, Zn^2+^ and Cu^2+^	Through skeletal interoception	Increase bone formation	^1^
Conductive hydrogel scaffolds	BP and polydopamine	Enhance the electrical conductivity of the hydrogels and improves the cell migration	Promote the differentiation of MSCs into neural-like cells	^64^
GelMA and SilMA bioprinted constructs	SCs-exos	Promote innervation, vascularization, and osteogenesis	Improve bone formation	^11^
Small extracellular vesicles	Small extracellular vesicles from injured neurons	Target osteoprogenitor	Stimulate bone formation	^12^
β-tricalcium phosphate scaffold	Sensory nerve and BMSCs	Improve the expression of CGRP and GAP43	Regulate the bone formation and the blood flow	^66^

## Challenges and future prospects

The expanding field of skeletal interoception has revealed the critical role of the nervous system in regulating bone metabolism, emphasizing the intricate connection between skeletal health and overall body metabolism. This system involves sensory nerves within the bone that transmit mechanical and biochemical signals to the CNS, enabling the brain to regulate bone remodeling and metabolic processes. Mechanistic studies have highlighted essential pathways, such as the PGE2-EP4 receptor axis, which plays a key role in neuroendocrine signaling that influences both bone formation and fat metabolism. This pathway illustrates how mechanical stimuli from bones are processed by the CNS to maintain bone health. Despite these advances, the precise molecular and cellular mechanisms involved in skeletal interoception remain only partially understood, necessitating further investigation.

Key areas for future research include identifying the specific sensory receptors present in bone tissue and the neural pathways they activate. For example, studies have shown that parathyroid hormone treatment can influence sensory nerve innervation in porous bone endplates, highlighting a potential neural mechanism in bone metabolism regulation. Understanding how the CNS processes these interoceptive signals is essential for constructing a systemic response to skeletal changes. By dissecting the neural circuits and brain regions involved in this process, researchers can gain insights into how skeletal information is integrated with other physiological signals to maintain bone and overall health.

The mechanistic understanding of skeletal interoception holds significant potential for developing new therapeutic strategies. Modulating neural pathways and sensory feedback could lead to innovative treatments for bone disorders like osteoporosis and fractures, as well as for metabolic conditions. Targeting pathways such as PGE2-EP4 or utilizing biomaterials to modulate neural feedback may enhance bone regeneration and repair. Given the impact of skeletal interoception on broader metabolic processes, understanding these pathways could lead to comprehensive therapeutic approaches that address both bone and metabolic diseases. While notable progress has been made, much remains to be explored, and further research promises to enhance our understanding of bone physiology and open new avenues for treating bone and metabolic disorders.

The rapid advancements in biomedical research are shedding light on the complex mechanisms of skeletal interoception, aided by the introduction of cutting-edge technologies like assembloids and Artificial Intelligence (AI).^68,69^ These innovative approaches are not only offering new pathways to deepen our understanding of the interactions between the skeletal system and the nervous system but are also paving the way for breakthroughs in biomedical research and clinical therapies.

**Fig. 4:**
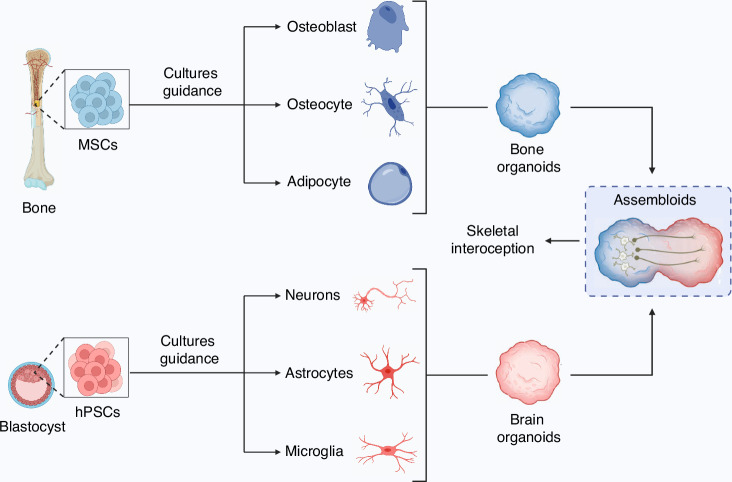
Illustration of skeletal interoception and assembloids

Assembloids are advanced self-organizing systems composed of multiple organoids or specialized cell types, offering a unique platform for simulating the interaction between different organ systems, including the skeletal and nervous systems.^70,71^ In the context of skeletal interoception, constructing assembloids that mimic the cellular and structural components of both bone and neural tissues enables researchers to directly observe the communication pathways between these two systems (Fig. 4).

Advances in materiobiology and stem cell technologies are poised to further enhance the construction of assembloids that accurately replicate the bone matrix, incorporating key cellular components such as osteoblasts, osteoclasts, and osteocytes, alongside corresponding neural elements like sensory neurons and glial cells.^72^ These next-generation models hold the potential to simulate how mechanical stress or biochemical signals from bone cells are transmitted via sensory neurons to the CNS. Such developments will likely offer unprecedented insights into the neuronal feedback mechanisms that regulate bone homeostasis and regeneration, ultimately paving the way for more effective therapeutic strategies.

In this system, assembloids can help explore how certain neuropeptides, neurotransmitters, or hormones, such as PGE2 or NPY, modulate the interoceptive feedback loop. Moreover, the integration of advanced imaging techniques, like live-cell fluorescence microscopy, with assembloid platforms enables continuous monitoring of neural responses to external stimuli, such as mechanical forces or injury. This capability makes assembloids invaluable for understanding the complex interactions between sensory neurons and osteocytes, particularly in the context of disease models, such as osteoporosis or osteoarthritis. By constructing these bone-neural assembloids, researchers can also explore how systemic metabolic states, such as obesity or diabetes, influence skeletal interoception and, consequently, bone health. For example, they can simulate how adipokines or glucocorticoids affect the bone-neural crosstalk, leading to potential breakthroughs in targeted therapies for metabolic bone disorders.

Thus, assembloids provide a dynamic model that bridges the gap between cellular-level interactions and systemic responses, allowing for a more nuanced understanding of skeletal interoception and its role in maintaining bone health and overall metabolism. Their application in drug screening or personalized medicine further underscores their potential in clinical translational research.

AI, a field rooted in computer science, aims to mimic human cognitive functions, integrating disciplines such as computer science, mathematics, and psychology.^73^ Since its inception in the 1950s, AI has evolved to find applications in numerous sectors, including biomedicine.^74^ In biomedical research, AI has the potential to revolutionize personalized medicine, drug discovery, and healthcare accessibility.^75^

**Fig. 5:**
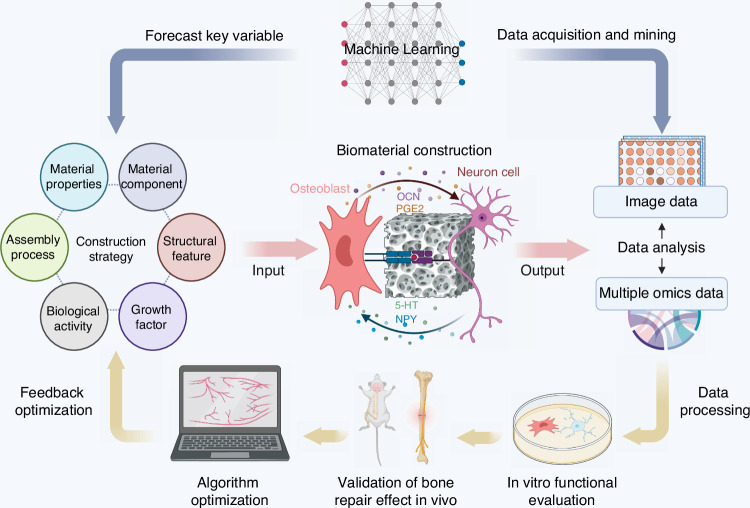
Integration of machine learning in biomaterial design for skeletal interoception

In skeletal interoception research, AI plays a pivotal role in optimizing biomaterial designs that target the neural regulation of bone regeneration (Fig. 5). For example, AI-driven algorithms can integrate data from various experimental sources, such as bone tissue biomechanics, neural electrophysiology, and cell-matrix interactions, to predict how different biomaterial compositions will interact with osteoblasts and neural cells. This allows for the precise tailoring of biomaterials that facilitate the recruitment of sensory neurons and enhance their role in bone formation and healing.

One specific application of AI is the use of predictive modeling to forecast the optimal conditions for bone-neural integration. By analyzing data from in vitro models, such as assembloids, and in vivo animal studies, AI can predict which combinations of neurotrophic factors, cytokines, and biomaterial properties will most effectively enhance bone regeneration. This predictive capacity is invaluable for reducing the time and cost of developing new bioactive scaffolds or drug delivery systems that target neural pathways involved in bone metabolism.

Additionally, AI’s ability to analyze multi-omics data, including transcriptomics and metabolomics, helps elucidate the molecular mechanisms underlying skeletal interoception. For example, AI algorithms can identify key genes or signaling pathways activated during bone remodeling in response to neural stimuli, which may lead to the discovery of novel therapeutic targets. These insights are critical for designing biomaterials that not only promote bone growth but also modulate neural inputs to enhance bone homeostasis.

The integration of AI into skeletal interoception research enables a more holistic approach to understanding the complex neural-regulated mechanisms that govern bone health. By leveraging its capacity for data analysis and predictive modeling, AI facilitates the development of next-generation biomaterials and therapeutic strategies that promise to significantly enhance bone repair and regeneration through the modulation of neural pathways.

In summary, the combined use of assembloids and AI provides new tools and methodologies for a deeper understanding of skeletal interoception. This integration is instrumental in revealing the complex interactions between the skeletal and nervous systems and in developing new therapeutic approaches for bone-related disorders. With the continuous evolution and refinement of these technologies, we can anticipate their increasingly significant role in bone health and disease treatment in the near future.

## Conclusions

In conclusion, the review highlights the significant role of skeletal interoception in bone homeostasis and the innovative potential of related biomaterials in treating bone disorders. Additionally, this review underscores the pivotal role of PGE2 within the framework of skeletal interoception for maintaining bone homeostasis. The targeted manipulation of PGE2 pathways, alongside the strategic incorporation of bioactive elements like divalent metal cations into biomaterials, offers a path toward enhancing bone regeneration. While these advancements are promising, comprehensive research and clinical trials are essential to establish their safety and efficacy. This burgeoning field stands at the forefront of orthopedic research, offering new therapeutic possibilities for improving the quality of life for those with bone-related conditions.
